# Psychological health in COVID-19 patients after discharge from an intensive care unit

**DOI:** 10.3389/fpubh.2022.951136

**Published:** 2022-08-12

**Authors:** Valeria Carola, Cristina Vincenzo, Chiara Morale, Massimiliano Pelli, Monica Rocco, Giampaolo Nicolais

**Affiliations:** ^1^Department of Dynamic and Clinical Psychology, and Health Studies, Sapienza University of Rome, Rome, Italy; ^2^Department of Clinical and Surgical Translational Medicine, Sapienza University of Rome, Rome, Italy

**Keywords:** SARS-CoV 2, COVID-19, clinical psychology, K10, perceived stress, state anxiety, intensive care unit

## Abstract

Along with physical changes, psychological changes are detectable in patients with COVID-19. In these patients, the stressful experience of intensive care unit (ICU) hospitalization may aggravate psychological conditions. Our study examines the short- and long-term psychological consequences of COVID-19 in ICU patients. COVID-19 patients completed the self-rating questionnaires *Kessler 10 Psychological Distress Scale* (K10), *Perceived Stress Scale-10* (PSS), *Impact of Event Scale Revised (*IES-R), and *Post-traumatic Growth Inventory (*PTGI) and were clinically interviewed 1 and 6 months after discharge. Altered behavioral-psychological symptoms and patients' strategies (adaptive vs. maladaptive) for *coping* with stress during and after hospitalization were coded during clinical interviews. Between 20 and 30% of patients showed moderate symptoms of depression or anxiety and perceived stress 1 and 6 months after discharge. Sleep problems, difficulty concentrating, confusion in placing events, and fear of reinfection were observed in many (6–17%) patients. At 6 months, only 7% of patients showed PTSD symptoms, and 50% showed post-traumatic growth in the “appreciation of life” sub-scale. Finally, 32% of subjects were classified as “maladaptive coping patients,” and 68% as “adaptive coping patients.” Patients who adopted “adaptive” coping strategies showed significantly lower levels of anxious-depressive symptoms and perceived stress when compared to subjects with “maladaptive” strategies at both time points. Coping strategy had no effect on PTSD symptoms or post-traumatic growth at 6 months. These findings clarify the short- and long-term psychological effects of intensive care due to COVID-19 infection and demonstrate that patient characteristics, particularly strategies for coping with stress, seem to play a critical role in psychological outcomes.

## Introduction

The novel coronavirus 2019 (COVID-19) pandemic has generated worldwide alarm. COVID-19 infection causes respiratory disease ranging from mild—or pauci-symptomatic—to fatal (1). Since its outbreak, COVID-19 has infected more than 300 million people and resulted in over 5 million deaths (2). Italy was one of the first countries to be severely impacted by COVID-19; more than 8 million confirmed cases and more than 130 thousand deaths were recorded in Italy as of December 2021 (2, 3).

COVID-19 affects both physical and psychological health. The post-acute phase of disease is often characterized by physical and psychological sequelae ranging from respiratory difficulty, cardiovascular abnormality, and prolonged infirmity to neurological and psychological (including cognitive and behavioral) complications (1). Psychological stressors, such as fear of illness, uncertainty about the future, traumatic memories of severe illness, and social isolation, may foster psychopathological outcomes, which, in turn, may worsen a patient's general medical condition (3, 4). Furthermore, studies suggest that coronaviruses can indirectly induce psychopathological sequelae *via* an immune response as well as by direct viral infection of the central nervous system. COVID-19 can cross the blood-brain barrier and infect the central nervous system (CNS), resulting in both short- and long-term neurological and neuropsychological sequelae (5–11). There is increasing evidence that while people with mild or moderate COVID-19 infection generally develop only respiratory symptoms, some patients with severe infection also develop neurological conditions like confusion, stroke, and even infectious toxic encephalopathy and viral encephalitis (12).

COVID-19 has a significant emotional impact on patients due to both the characteristics of the virus itself and its psycho-physical-social consequences. COVID-19 survivors often develop psychiatric distress such as insomnia, along with anxious, depressive, and even post-traumatic psychological reactions (11, 13, 14). Younger age, chronic disease, or a history of psychiatric illness may contribute to the development of depressive and anxious symptoms during the pandemic, while more social support, including physical and psychological assistance, is correlated with lower stress levels (13, 15–20).

In 5–11% of cases, COVID-19 infection causes medical complications, chiefly acute respiratory failure, that necessitate hospitalization in an intensive care unit (ICU) (21); treatment of ARF (22) often involves mechanical ventilation. ICU care can have a major impact on the psychological wellbeing of patients (23). The ICU environment can be stressful, with the noise of medical devices, constant lighting, ongoing alarms, and staff working under pressure. Some studies suggest that sounds alone can contribute to sleep and mood disturbances (24, 25). ICU patients have limited freedom of movement, and especially patients in need of ventilation support may have difficulties communicating. During invasive and prolonged medical interventions, patients under sedation often have impaired perception, and they may experience altered mental states including delirium. Such experiences in an ICU may themselves become a specific risk factor for the development of psychopathology and for a reduction in psychological wellbeing and quality of life. Studies suggest that patients are at a heightened risk for experiencing psychological symptoms during and following an ICU stay (26, 27). During the first year of recovery after an ICU stay, approximately one third of survivors experience symptoms of anxiety and depression, and about a fifth experience clinically important symptoms of PTSD (26, 28–33). In a systematic review (28), the median point prevalence of questionnaire-assessed substantial PTSD symptoms was 22%, and the median point prevalence of clinician-diagnosed PTSD was 19% assessed from ≈6 weeks to 7 years, though most studies had PTSD assessments within the first year post-ICU. Pre-ICU psychopathology, greater in-ICU benzodiazepine administration, and post-ICU memories of frightening and/or psychotic experiences in the ICU might predict the onset of post-traumatic syndrome after discharge (28, 34). While it is well known that ICU admission can result in PTSD, literature on the short- and long-term consequences of ICU admission specifically for COVID-19 is lacking.

Traumatic experiences like ICU hospitalization can also lead to positive developments. This phenomenon is known as post-traumatic growth (PTG), defined as the subjective experience of a significantly positive change for an individual following a major life crisis. PTG can follow many different types of traumatic event, such as bereavement (35), combat (36), or cancer (37).

Patients' coping strategies during adaptation to trauma may have a dramatic impact on their general recovery. Adaptive coping strategies, i.e., problem-centered strategies that help the individual openly face and internalize the traumatic event, correlate with better outcomes (38–40). On the other hand, maladaptive coping strategies, aimed at the reduction of tension *via* the activation of specific defensive mechanisms, are likely to worsen the patient's current conditions and prognosis (40–43).

Literature on the psychopathological sequelae of COVID-19 patients after ICU hospitalization is still largely lacking. Longitudinal data at 3 months after discharge are rare (44–46), and, to our knowledge, longer-term data are non-existent.

We found little evidence of published studies related to PTG in COVID-19 patients (47–49) particularly with samples of COVID-19 patients after discharge from the ICU (49).

Similarly, we found no studies on the use of adaptive vs. maladaptive coping strategies in COVID-19 patients after ICU hospitalization.

We hypothesize, first, that the experience of COVID-19 infection may be a risk factor for the development of anxious, depressive, and PTSD symptoms; second, that post-traumatic growth may occur following recovery from COVID-19; and third, that psychopathological outcomes may be moderated by coping strategies adopted by the patients.

## Materials and methods

### Participants

All patients (*N* = 71) who were infected with COVID-19 and admitted to the ICU of the Sant'Andrea University Hospital in Rome (73.5% men, 26.5% women), between November 2020 and May 2021, were included in this study. Patients completed self-assessment questionnaires and were clinically interviewed 3 months (Timepoint 1, T2) and 5–6 months (Timepoint 2, T2) after discharge. Psychological interviews were included in a general medical screening after discharge. The subjects' mean age was 60 years, and the age range was 34–85 years.

Prior to enrolment, all participants were given a complete description of the study and provided written informed consent. The patients were interviewed and screened for psychological symptoms using questionnaires. The study was approved by the Ethical Committee of the Department of Dynamic and Clinical Psychology, Sapienza, University of Rome (Prot. n. 0000144).

### Psychometric tools

Questionnaires were administered online or, for patients who had difficulty with digital tools, face-to-face before the interviews. At the first time point, 1–3 months after discharge, patients completed the Kessler 10 Psychological Distress Scale (K10) and Perceived Stress Scale (PSS) questionnaires. At the second time point, 5–6 months after discharge, the same patients completed the K10 and PSS questionnaires again and, in addition, the Post-Traumatic Growth Inventory (PTGI) and Impact of Event Scale Revised (IES-R) questionnaires. At the second time point, 10% of patients in the first sample were excluded from the second interview because they had begun psychotherapy.

#### Kessler 10 psychological distress scale

K10 (50), a 10-item questionnaire, provides a global measure of distress experienced in the previous 4 weeks. We used the validated Italian translation (51). Each item is scored on a 5-point Likert scale: 1 (“never”), 2 (“rarely”), 3 (“some of the time”), 4 (“most of the time”), or 5 (“all of the time”). To be consistent with previous validation studies (50, 52), patients who scored between 20 and 24 were considered mildly distressed, and patients who scored between 27 and 40 were considered highly distressed.

#### Perceived stress scale-10

PSS (53), a 10-item questionnaire, measures the degree to which one perceives aspects of one's life as uncontrollable, unpredictable, and overwhelming. Participants are asked to respond to each question on a 5-point Likert scale ranging from 0 (never) to 4 (very often), indicating how often they have felt or thought a certain way within the past month. Scores range from 0 to 40, with higher composite scores indicative of greater perceived stress. Patients were considered to have intermediate perceived stress if they scored between 18 and 26, whereas they were considered to have high perceived stress if they scored between 27 and 40. The PSS possesses adequate internal reliability (53).

#### Post-traumatic growth inventory

PTGI (54) is a 21-item inventory that assesses the positive psychological change that may occur after a traumatic experience. We used the validated Italian translation (55). Participants are asked to respond to each statement on a 6-point Likert scale ranging from 0 (“I did not experience this change as a result of my crisis”) to 5 (“I experienced this change to a very great degree as a result of my crisis”), with intermediate scores of 1 (“a very small degree”), 2 (“a small degree”), 3 (“a moderate degree”), and 4 (“a great degree”). The PTGI assesses patient growth on five sub-scales: relating to others, new possibilities, personal strength, spiritual change, and appreciation of life. Patients' scores were compared to scores obtained by an Italian normative sample (55). Mean of the normative reference sample was for “appreciation of life” 7.66 ± 4.37, for “personal strength” 9.48 ± 5.64, for “relating to others” 14.12 ± 9.13, for “new possibilities” 10.38 ± 7.07, and for “spiritual change,” 3.33 ± 3.36. The test-retest reliability (alpha) of the PTGI is 0.71 and its internal consistency is 0.90 (55).

#### Impact of event scale revised

IES-R (56), a 22-item questionnaire, assesses the magnitude of symptomatic response in the past 7 days to a specific traumatic life event. This version of the IES comprises three dimensions: avoidance, intrusion, and hyperarousal. We used the Italian validation (57). Participants are asked to report their degree of distress during the past 7 days on a 5-point Likert scale: 0 (not at all), 1 (a little bit), 2 (moderately), 3 (quite a bit), or 4 (extremely). Given the timing of PTSD onset assessable 1 month after the traumatic event (58), we assessed the presence of post-traumatic symptoms only at the second time point.

### Clinical assessment

Clinical interviews were conducted within a medical screening process prepared by the ICU and investigated the following areas:

- general anamnestic information
- sleep quality before, during, and after hospitalization
- post-traumatic symptomatology
- spiritual faith and its possible supporting role for the patient
- memories and experiences of hospitalization
- current psychological state.

Frequency tables of reported behavioral-psychological symptoms were developed from the content of the interviews.

Adaptive and maladaptive coping strategies employed by the patient during and after hospitalization were investigated during the clinical interviews. Coping strategies are behaviors implemented by individuals to deal with stressful or traumatic situations. In accordance with the literature (38, 59), we define adaptive coping strategies as problem-centered strategies (such as active coping, planning, and social support). Maladaptive coping strategies are strategies aimed at reducing tension (such as avoidance, denial, and emotional release). Assessment of coping strategies from the content of the interviews was performed independently by four different certified psychologists. Indicators of post-traumatic growth were also assessed from the interviews.

### Statistics

Count data were expressed as frequency and percentage. Measurements were described by the mean and standard deviation. Repeated-measures analyses of variance (RM-ANOVA) were performed to assess the long-term effects of ICU admission due to COVID-19 on anxious-depressive symptoms (from the K10) and stress-related variables (from the PSS). One-way analyses of variance (ANOVAs) were performed to evaluate the impact of coping strategy on anxious-depressive and PTSD symptoms (from the K10 and IES-R), perceived stress levels (from the PSS), and post-traumatic growth (from the PTGI). Significant RM-ANOVAs and ANOVAs (*P* < 0.05) were followed by *post-hoc* comparisons using Duncan's test. Statistical analyses were carried out using Statistica, version 12.0 (StatSoft, Tulsa, OK, USA).

## Results

### Anxious-depressive symptoms, perceived stress levels, and behavioral symptoms

**Figure 1:**
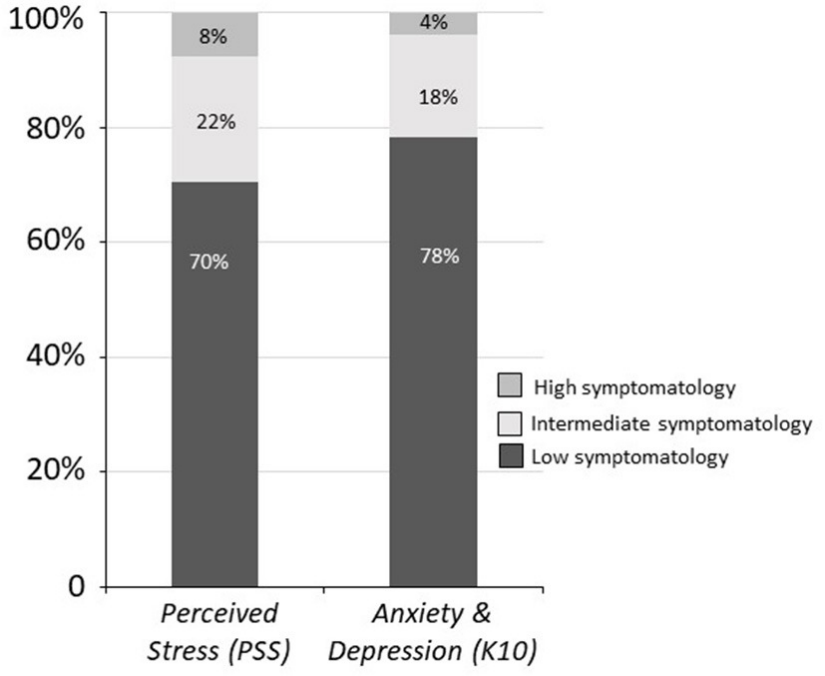
Frequencies of COVID-19 patients showing high, intermediate, and low symptomatology on the K10 and PSS (anxious-depressive symptoms and perceived stress) at T1.

To assess the levels of anxious-depressive symptoms and perceived stress in COVID-19 patients at the first time point, the frequencies of scores on the K10 and PSS were evaluated. Twenty-two percent of patients exhibited medium to high levels of anxious-depressive symptoms (sample mean ± SD: 16.85 ± 6.25; Figure 1). A larger percentage (30%) of patients showed medium to high levels of perceived stress (11.48 ± 9.32; Figure 1). Based on questionnaire results and each patient's psychological condition according to the clinical interview, 10% of patients were referred for psychotherapy and excluded from the assessment of psychological symptoms at the second time point.

**Figure 2:**
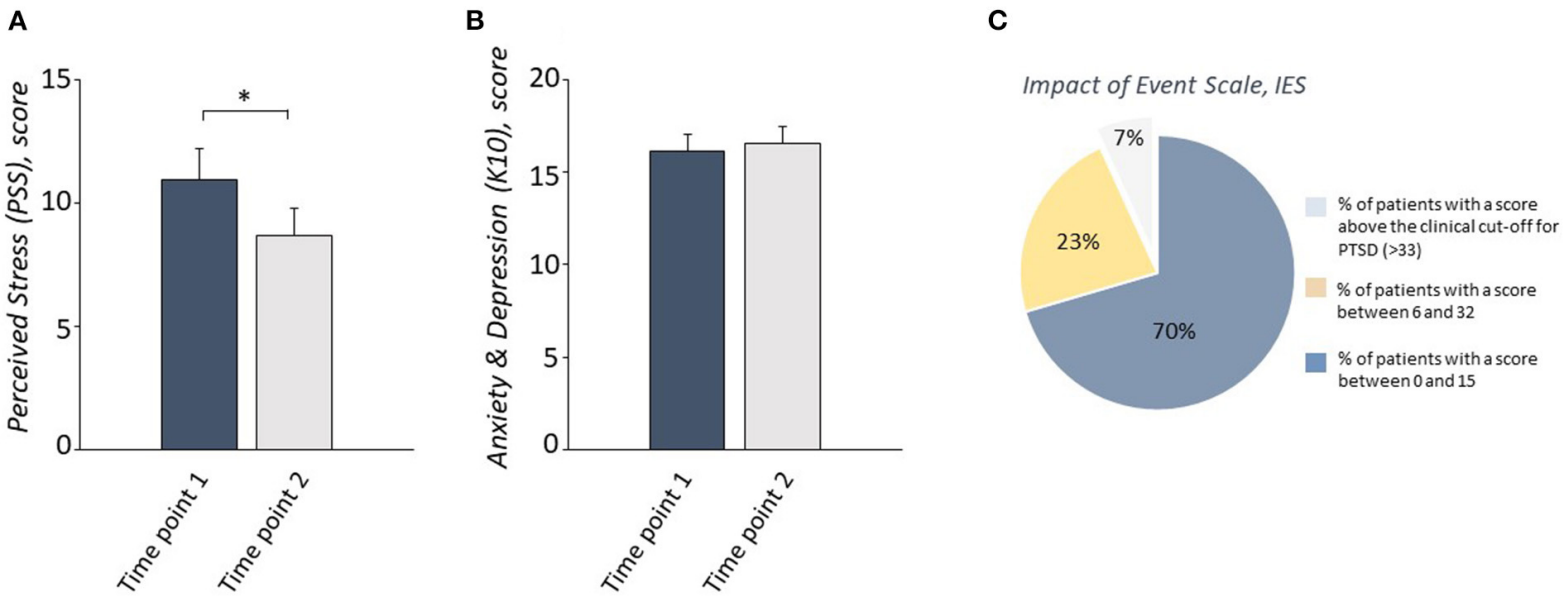
A reduction of the perceived stress levels **(A)**, but no change in anxious-depressive symptoms **(B)**, measured respectively by PSS and K10, was observed in COVID-19 patients between Time point 1 and Time point 2. 70% of patients obtained a score between 0 and 15, 23% obtained a score between 16 and 32, and only 7% of patients scored above the clinical cut-off for PTSD symptoms measured by IES-R at Timepoint 2 **(C)**. *P < 0.05.

To evaluate whether levels of anxiety-depression and of perceived stress had changed between the first and second time points, two repeated-measure ANOVAs were performed on the scores from the K10 and the PSS. No significant change in the level of anxious-depressive symptoms was detected between time points (Figure 2). Time had a statistically significant effect on PSS scores; levels of perceived stress were lower at time point 2 than at time point 1 (Figure 2).

The frequency of other behavioral symptoms was also recorded during the clinical interviews at the first time point. Specifically, 17% of patients reported sleep problems, 9% reported active inhibition of ICU memories, 7% reported confusion in temporally placing events, 7% reported short-term memory problems, 14% reported concentration problems, and 6% reported fear of being infected again (Table 1).

**Table 1:** Frequency of behavioral symptoms recorded during the clinical interviews at time point 1.

**Sympthoms post ICU**	**% Patients**
Sleep problems	17
Active inhibition of ICU memories	9
Confusion in temporally placing events	7
Memory problems (short-term)	7
Concentration problems	14
Fear of being infected by Covid again	6

### Long-term PTSD symptoms and post-traumatic growth

In order to detect PTSD symptoms at the second time point, the frequencies of scores on the IES-R questionnaire were recorded. Only 7% of patients showed a score above the clinical cutoff of 33; the majority of the sample showed no symptoms or moderate symptoms (total IES-R = 12.09 + 12.33; Table 2, Figure 2).

**Table 2:** Descriptive statistics of the IES-R and PTGI sub-scales (mean + st.dev.).

**Measure**	***M***	***SD***
PTGI- Relating to others	11.61	9.81
PTGI-New possibilities	7.02	7.31
PTGI-Personal Strength	6.82	6.13
PTGI-Spiritual Change	2.59	3.39
PTGI-Appreciation of Life	6.75	4.69
IES- Avoidance	0.48	0.57
IES-Intrusiveness	0.66	0.68
IES-Iperarousal	0.50	0.57

**Figure 3:**
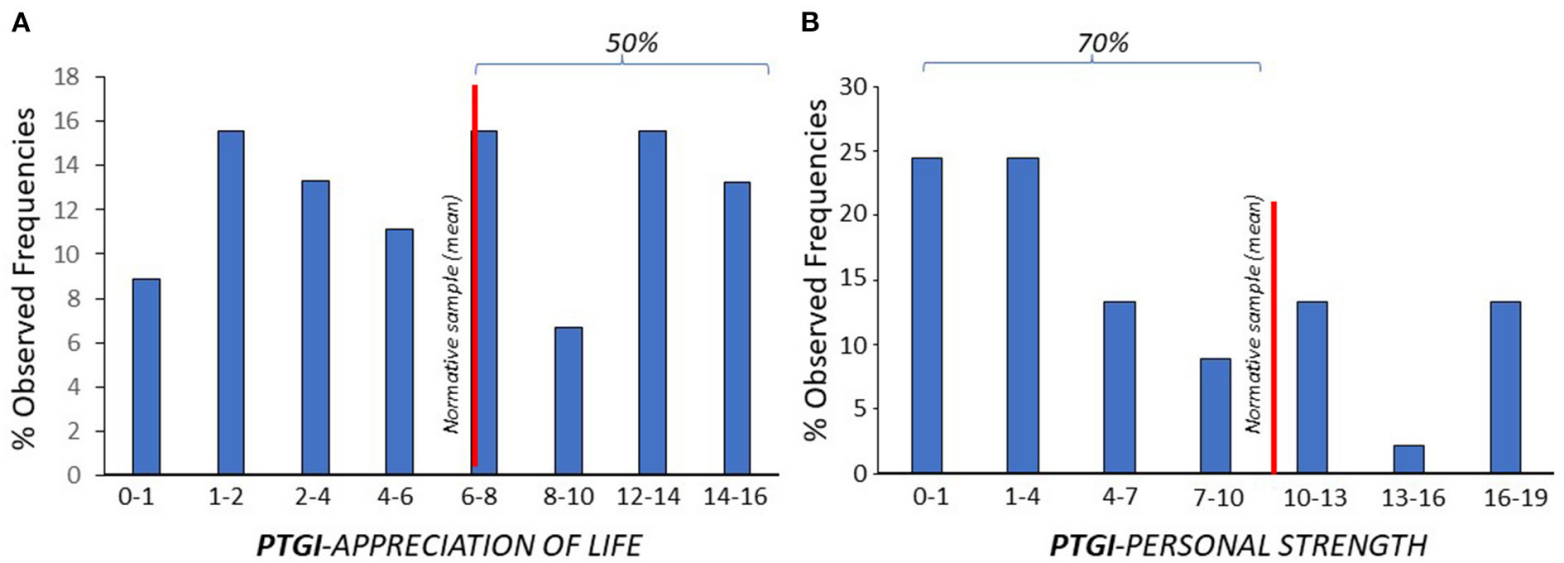
The fraction of patients who scored (% observed frequencies) at or below the normative reference sample (red line) was 48% for “appreciation of life” (**A**), and 68% for “personal strength” (**B**) subscales.

The presence of PTG on each of the PTGI sub-scales was evaluated at the second time point (Table 2, Figure 3). The fraction of patients who scored at or below the normative reference sample on each sub-scale was 48% % for “appreciation of life,” 68% for “personal strength,” 66% for “relating to others,” 73% for “new possibilities,” 66% for “spiritual change.”

### Impact of active vs. passive coping strategies on PSS, K10, IES-R, PTGI scores

**Figure 4:**
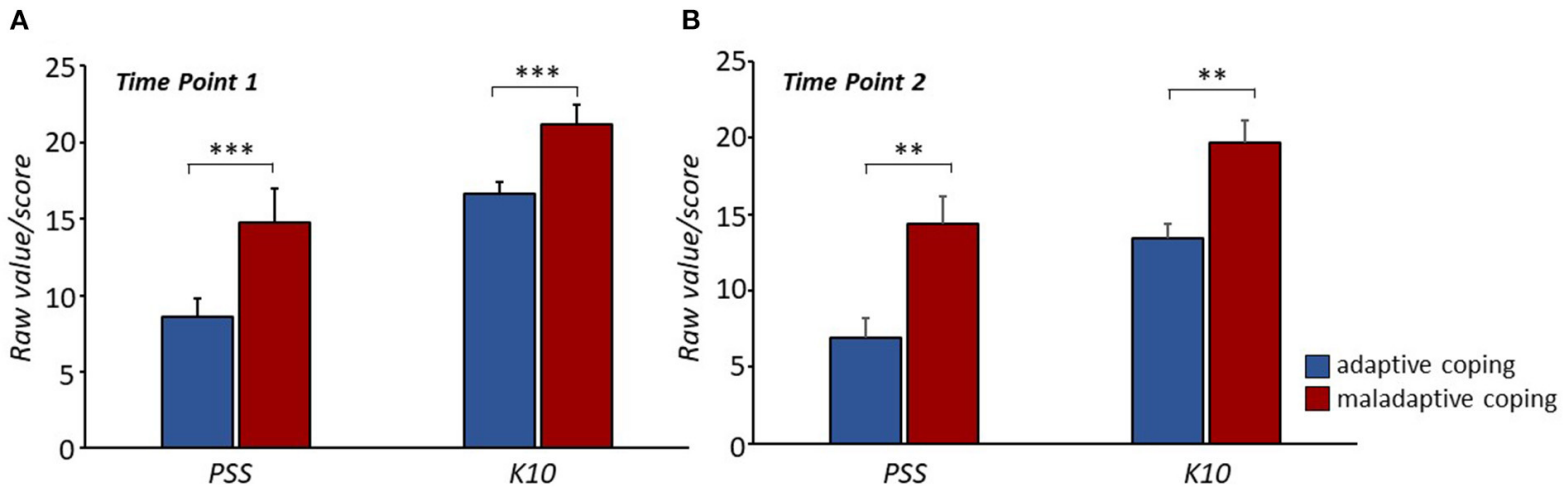
Significant lower perceived stress levels and anxious-depressive symptoms were observed in “adaptive coping patients” compared to “maladaptive coping patients” at both time points (**A, B**). **P < 0.01, ***P < 0.001.

Qualitative analysis of clinical interviews at the first time point classified 32% of patients as “maladaptive coping patients” (8M and 3F; age 63.20 ± 11.29) and 68% as “adaptive coping patients” (22M and 5F; age 60.92 ± 10.66). Four ANOVAs were performed to assess whether the coping strategy was associated with anxious-depressive symptoms and levels of perceived stress at each of the two time points. Coping strategy had a statistically significant effect on both scores at both time points; “adaptive coping patients” showed significantly lower levels of anxious-depressive symptoms and perceived stress than “maladaptive coping patients” at both time points (perceived stress, time point 1: *F*_(1, 65)_ = 11.91, *p* < 0.001; perceived stress, time point 2: *F*_(1, 37)_ = 7.85, *p* = 0.008; anxiety-depression, time point 1: *F*_(1, 65)_ = 21. 51, *p* < 0.001; anxiety-depression, time point 2: *F*_(1.37)_ = 8.90, *p* = 0.005; Figure 4).

ANOVAs were performed to assess whether coping strategy was associated with PTSD symptoms and PTG levels at the second time point. Coping strategy showed no significant effect on PTSD or any of the PTG sub-scales.

### Qualitative analysis of clinical interviews

The perception of post-traumatic growth, along the six dimensions of the PTGI, was investigated through qualitative analysis of the clinical interviews. The subjects' accounts provided an overview of the type of post-traumatic growth present in the sample and its characteristics.

#### Relating to others

Growth in “relating to others” was one of the clearest trends that emerged in clinical interviews. “Relating to others” refers to the propensity of survivors of a traumatic event to talk to family, friends, or fellow survivors, with whom survivors might feel a sense of closeness and connection. Growth along this dimension underscores the subject's greater sensitivity to interpersonal relationships and thus greater perception of appreciating and valuing them. For example, one of the interviews reported,

(…) He acknowledges a high level of apprehension toward his wife and son after hospitalization and acknowledges that at times this is exacerbated by the lived experience (…) Reports that after discharge he is reconsidering priorities in his life, giving more space to his marital relationship and with his son (…).

#### Appreciation of life

“Appreciation of life” relates to how trauma clarifies what is truly important in the survivor's life. Having experienced a traumatic event can lead the survivor to reconsider the value of life, as well as the precarious balance between life and death. For example, one interview reported,

(…) He can't wait to get back to work and wants to devote himself body and soul to patients, especially those in intensive care. He says he knows what it feels like in those conditions and how important even a caress or an extra word is in those places, so he wants to commit himself to this field. He feels that this has been an important life experience that has changed him for the better (…)

#### New possibilities

“New possibilities” was a prominent theme in the clinical interviews. “New possibilities” refers to the readiness of the survivor to experience new life scenarios following the traumatic event. The traumatic event and the experience of survival become the engine that pushes the survivor to live new realities, seek new experiences, and pursue new interests. For example,

(…) If she had to go back, she would seriously think about being a nurse. She ended up being so motivated that we even talked about the possibility for her to volunteer in hospitals in the future (…). She feels it is her mission to tell everyone about what she went through there and the humanity she found, so that everyone can appreciate what the doctors and nurses at ICU do for us.

#### Personal strength

“Personal strength” was the area of post-traumatic growth most evident in the clinical interviews. “Personal strength” refers to the development of greater self-efficacy and capacity. The survivor of the traumatic event may feel greater confidence in their own actions and wisdom and therefore may feel more able of dealing with future events. In the interviews, participants reflected on their increased perception that they could cope with future challenges.

(…) He feels that the ICU experience may have improved him because now when he faces a problem, he realizes that he is calmer because he knows that a solution can always be found (…)

#### Spiritual change

“Spiritual change” was not a prominent aspect of the post-traumatic growth experienced by participants. This dimension concerns an individual's connection to nature, to others, and to the world and the individual's understanding and acceptance of him/herself and others.

## Discussion

Despite growing interest in the effects of the COVID-19 pandemic and of infection on mental health (60–62), many unknows remain. This study investigated the short- and long-term psychological effects of COVID-19 infection on patients who experienced acute illness and ICU admission. Among our patients, 22% exhibited moderate anxious and depressive symptoms, and 30% exhibited medium to high levels of perceived stress ≈1 month after discharge from the ICU. Levels of anxious and depressive symptomatology remained stable, while perceived stress levels in this sample decreased by ≈6 months after discharge. These findings are in line with many studies that suggest that anxious and depressive symptoms result from hospitalization due to COVID-19 (13, 43, 63–65). A meta-analysis by Saidi et al. (66) found prevalence levels for symptoms of depression and anxiety at 45 and 47%, respectively, in hospitalized COVID-19 patients. Other studies have reported rates ranging from 18 to 30% within the first 3 months after discharge (44–46). Deng et al. (67) and Moayed et al. (68) found a prevalence of 46.6% for elevated perceived stress in patients infected with COVID-19. SARS and MERS patients admitted to the ICU experienced similar psychological distress that persisted even 6 months after discharge (15).

Our interviews revealed other relevant behavioral symptoms, including sleep disturbances, concentration problems, active inhibition of ICU memories, confusion in the temporal placement of events, short-term memory problems, and fear of re-infection. The presence of sleep-related disorders seems to be in line with data from previous studies (66) in which the prevalence of sleep disorders was 34% among COVID-19 patients. Consistent with our study, Poyraz et al. (45) found that a notable percentage of COVID-19 patients also reported behavioral symptoms after recovery, such as sleep disturbance and difficulty concentrating in 38.8 and 15% of the sample, respectively. A qualitative study (68) of patients admitted to the ICU for other medical causes found an absence of ICU-related memories at 3 months post-discharge, likely because the patients had been sedated. In our study a substantial percentage of COVID-19 patients complained of active memory inhibition in the ICU, even though they had been awake and conscious during hospitalization.

The experience of being hospitalized for COVID-19 has characteristics that make it a risk factor for the development of PTSD (28, 33). However, ICU admission for COVID-19 appears to be associated with relatively low prevalence rates of PTSD. In our sample, only 7% of patients reported PTSD symptoms. In a meta-analysis, Nagarajan et al. (69) found a 16% prevalence of PTSD among patients with COVID-19 infection that led to acute illness. By contrast, for coronaviruses SARS and MERS, the prevalence of post-admission PTSD was 39% (15). For other medical causes, the prevalence of PTSD after ICU admission is 19–22%, higher than but comparable to that for COVID-19 (28).

The potential for psychological growth after trauma is less well studied than the more physiological consequences of trauma (70, 71) and may add an important perspective to current thinking about trauma. PTG can be transformative; in the face of emotionally overwhelming and stressful events, individuals can commit their resources and skills toward overcoming adversity and emerge with a perception of an improvement in themselves (72–74). This process in survivors of intensive care for COVID-19 has been studied little. Our results agree with previous work (47–49) that PTG can be significant in patients who have experienced intensive care for COVID-19, even if it is accompanied by moderate psychological distress. We found significantly higher “appreciation of life” but lower “personal strength” among our sample relative to the Italian normative sample. Less PTG in “personal strength” may make sense given the history of our sample. The feeling of helplessness and the lack of autonomy that ICU patients experienced during wakefulness may have contributed to lower self-efficacy, especially for managing future adverse events after hospitalization. The lower values of “personal strength” may also have been a consequence of “long COVID,” defined as the persistence of fatigue and residual respiratory symptoms after recovery (75, 76). Previous studies have shown positive associations between COVID-19-related concerns and PTG in samples of U.S. civilians and veterans (70, 77). Others have reported high rates of PTG, especially in “appreciation of life” and “relating to others,” in a sample of parents in Portugal during the pandemic (78). An assessment of PTG in hospitalized patients undergoing bone marrow and/or stem cell transplantation and palliative care (79, 80), found, as did our study, that “appreciation of life” was among the areas of most significant growth; the assessment also found no change in “personal strength.” Finally, a study conducted by Holtmaat et al. (81) on cancer patients showed that the most impacted domain was “relating to others.”

To investigate patient response to the experience of hospitalization in the ICU, we further assessed the coping strategies patients used during hospitalization and upon recovery. In our sample, 32% of subjects were classified as “maladaptive coping patients” while 68% were classified as “adaptive coping patients.” Patients who adopted “adaptive coping strategies” showed significantly lower levels of anxious-depressive symptoms and PSS, compared to subjects with “maladaptive strategies.” But coping strategy had no effect on PTSD symptoms or PTG levels. An association between adaptive coping style and lower risk of psychological distress has also been described in students during the COVID-19 pandemic (82). A lack of association between coping strategy and PTSD symptoms has been previously observed, as well (83). Although the relationship between coping strategies and PTG in COVID-19-infected populations is still not well established, our data appear to be at odds with recent research showing that coping strategies can influence PTSD and PTG in other contexts (49, 74, 84–87). Adaptive coping strategies, such as problem solving, were positively associated with high levels of PTG and negatively correlated with PTSD symptoms in military and civilian samples. Together, our findings and the wider literature suggest a complex relationship between coping strategy, PTG, and PTSD symptoms that should be further investigated.

## Limitations

Our sample was small and not representative; the characteristics of the sample itself and of the pandemic more generally did not allow for the selection of an ideal experimental sample. Because of sample size, we did not perform statistical analyses controlling for demographic factors such as age groups, gender, and level of education.

Second, in our study we did not perform an evaluation of risk factors for the development of anxiety, depressive or stress symptoms following COVID-19 infection and ICU hospitalization. In fact, longer-term hospitalization (88), female gender (14, 89–91), perception of low social support (14, 88), previous psychiatric problems (89) and low oxygen saturation (92) are associated with increased psychological distress at discharge.

Third, coping strategy was assessed on a clinical basis by four independent expert clinical evaluators and psychologists, rather than *via* a standardized questionnaire.

Fourth, the study was limited by specific characteristics of the sample and the absence of a control group. Because our sample included only patients who had been admitted to the ICU for COVID-19, it was not possible to compare them either to patients who had experienced only COVID-19 infection or to patients who had experienced an ICU admission independently of COVID-19.

Finally, the patients in our original sample who were most psychologically compromised at the first time point were referred, for ethical reasons, to psychotherapy services and thus were excluded from the second phase of assessment in which PTSD and PTG were investigated.

## Future perspectives

Future research should investigate whether the psychological effects of ICU hospitalization for COVID-19 resolve or persist over a longer timescale, especially in patients who used maladaptive coping strategies. Longitudinal studies should be performed at least 1 year after discharge from the ICU. In addition, comparisons should be made between patient groups like ours and (1) COVID-19 patients for whom ICU hospitalization was not necessary and (2) patients admitted to the ICU for other organic causes/pathologies. These controls would help clarify the independent effects of COVID-19 and ICU hospitalization.

## Conclusions

This study provided an in-depth look at the short- and long-term psychological effects of the experience of intensive care for severe COVID-19. Results indicated that patient characteristics and patient coping strategies may play a decisive role in psychological outcomes. Moreover, this study showed that survival of COVID-19 together with ICU hospitalization may foster positive psychological growth, as well.

## Data availability statement

The raw data supporting the conclusions of this article will be made available by the authors, without undue reservation.

## Ethics statement

The studies involving human participants were reviewed and approved by Ethical Committee of the Department of Dynamic and Clinical Psychology, Sapienza, University of Rome (Prot. n. 0000144). The patients/participants provided their written informed consent to participate in this study.

## Author contributions

VC, CV, MR, MP and GN design the study and collected the data. VC, CV, and CM analyzed the data and designed the figures/tables. All authors wrote the manuscript. All authors contributed to the article and approved the submitted version.

## Conflict of interest

The authors declare that the research was conducted in the absence of any commercial or financial relationships that could be construed as a potential conflict of interest.

## Publisher's note

All claims expressed in this article are solely those of the authors and do not necessarily represent those of their affiliated organizations, or those of the publisher, the editors and the reviewers. Any product that may be evaluated in this article, or claim that may be made by its manufacturer, is not guaranteed or endorsed by the publisher.
